# The cost of lost productivity due to premature cancer-related mortality: an economic measure of the cancer burden

**DOI:** 10.1186/1471-2407-14-224

**Published:** 2014-03-26

**Authors:** Paul A Hanly, Linda Sharp

**Affiliations:** 1 National College of Ireland, Mayor Street, Dublin 1, Ireland; 2 National Cancer Registry Ireland, Cork Airport Business Park, Kinsale Road, Cork, Ireland

**Keywords:** Productivity costs, Years of life lost, Cancer, Economic burden, Ireland

## Abstract

**Background:**

Most measures of the cancer burden take a public health perspective. Cancer also has a significant economic impact on society. To assess this economic burden, we estimated years of potential productive life lost (YPPLL) and costs of lost productivity due to premature cancer-related mortality in Ireland.

**Methods:**

All cancers combined and the 10 sites accounting for most deaths in men and in women were considered. To compute YPPLL, deaths in 5-year age-bands between 15 and 64 years were multiplied by average working-life expectancy. Valuation of costs, using the human capital approach, involved multiplying YPPLL by age-and-gender specific gross wages, and adjusting for unemployment and workforce participation. Sensitivity analyses were conducted around retirement age and wage growth, labour force participation, employment and discount rates, and to explore the impact of including household production and caring costs. Costs were expressed in €2009.

**Results:**

Total YPPLL was lower in men than women (men = 10,873; women = 12,119). Premature cancer-related mortality costs were higher in men (men: total cost = €332 million, cost/death = €290,172, cost/YPPLL = €30,558; women: total cost = €177 million, cost/death = €159,959, cost/YPPLL = €14,628). Lung cancer had the highest premature mortality cost (€84.0 million; 16.5% of total costs), followed by cancers of the colorectum (€49.6 million; 9.7%), breast (€49.4 million; 9.7%) and brain & CNS (€42.4 million: 8.3%). The total economic cost of premature cancer-related mortality in Ireland amounted to €509.5 million or 0.3% of gross domestic product. An increase of one year in the retirement age increased the total all-cancer premature mortality cost by 9.9% for men and 5.9% for women. The inclusion of household production and caring costs increased the total cost to €945.7 million.

**Conclusion:**

Lost productivity costs due to cancer-related premature mortality are significant. The higher premature mortality cost in males than females reflects higher wages and rates of workforce participation. Productivity costs provide an alternative perspective on the cancer burden on society and may inform cancer control policy decisions.

## Background

Cancer is currently the leading cause of death in economically developed countries [1]. While advances in diagnosis and treatment over the past decades have resulted in improved survival rates in developed countries [2], future growth in new cancer cases is projected due to population growth and ageing [1].

To inform the setting of priorities for cancer control it is necessary to quantify the cancer burden. A variety of different metrics are available. One measure that has gained prominence recently is years of potential life lost due to premature cancer-related mortality [3]. While years of potential life lost – and other conventional measures such as numbers of incident cases and deaths, and age-standardised rates – are important indicators, they take an entirely public health perspective, focussing on the health-related impact or burden of cancer on society. Cancer also has an economic impact on society [4]. One important element of this economic impact is the cost of lost productivity due to cancer-related premature mortality.

A few studies have estimated the costs of lost productivity, but these have generally considered individual cancer sites (e.g. skin [5], breast [6], pancreas [7]). While providing a useful insight into the proportion of economic costs related to individual cancers, they fail to yield an estimate of the overall burden of cancer-related premature mortality on the economy. Also, cancer control initiatives are often site-specific so estimates of productivity losses associated with different cancers are needed to inform decisions about allocation of healthcare funding between initiatives. A very limited number of studies of productivity loss in multiple cancer sites exist, mainly from North America [8,9] and Asia [10]; estimates from Ireland are absent and those from European countries generally are limited.

The aim of this study was to estimate – for all cancers and the ten most common causes of cancer death in males and females – years of potential productive life lost (YPPLL) and premature mortality costs in Ireland. We also compare these indicators with numbers of deaths and age-standardised rates, to illustrate how each provides a different perspective of the cancer burden on society.

## Methods

### General approach

We used the human capital approach to estimate the value of productivity lost due to cancer-related premature mortality in Ireland. The human capital approach measures the value to society of potential productivity, in the form of output that is lost due to disease-related morbidity and mortality and is valued by the market wage. Specifically, we estimated YPPLL, separately for males and females, for all cancers combined and for each of the ten most common causes of cancer-related death in adults. YPPLL were then valued using wage rates as an approximation of foregone productivity as is usual in the human capital approach [11]. Since these estimates relate to lost time from market (i.e. employment-related) activities, in a scenario analysis, we also estimated costs for lost time from non-market activities such as household activities and caring, and valued these using the proxy good approach which applies the value for an equivalent service provided in the market to the non-market activity [12]. Costs are expressed in 2009 euros.

### Data sources

Numbers of deaths during 2005-2009 by 5-year age-group and sex between the ages of 15 and 64 were abstracted from the World Health Organization (WHO) Cancer Mortality Database for all cancers (International Classification of Diseases (ICD) 10 00-97, B21). Ethical approval was not required as the study was based on publically available data [13] on numbers of deaths from cancer in Ireland. Data was abstracted on the following sites: oesophagus (ICD10 15, males and females); stomach (C16, males and females); colorectal (C18-21, males and females); pancreas (C25, males and females); lung (C33-34, males and females); breast (C50, females); uterus (C53-55, females); ovary (C56 – females); prostate (C61, males); bladder (C67, males); brain & CNS (C70-72, males and females); non-Hodgkin’s lymphoma (C82-85, C96, males and females); and leukaemia (C91-95, males and females). Data on age- and gender-specific wages came from the National Employment Survey 2009 [14] and data on age and sex-specific unemployment and labour force participation rates were abstracted from the Quarterly National Household Survey [15]. Future wage growth was approximated by forecast gross domestic product (GDP) growth for Ireland [16].

As regards non-market activities, time spent on household activities and caregiving amongst the general population were sourced from an Irish time-use survey [17]; this required the assumption that the time spent on these tasks was the same among people with cancer as the general population. Wages for household activity and caregiving were derived from Hanly et al. [12].

### Estimation methods

Numbers of deaths were converted into rates using population estimates from the Central Statistics Office, and standardised using the World Standard Population to provide World Age Standardised Rates (WASR; http://www-dep.iarc.fr/WHOdb/glossary.htm).

Estimation of YPPLL followed a methodology previously described [18]. To calculate YPPLL we disregarded any deaths, in children (<15) and beyond 64 years, thereby assuming all those working will retire at 65, the official pensionable age in Ireland in 2009. We assumed, for example, each death in the 55-59 age group was aged 57.5 at death; YPPLL for that death was therefore 7.5 years (65-57.5). Then, YPPLL for each death were summed across age-groups, by sex, and by cancer site.

Valuation of premature mortality costs involved multiplying, for each death, YPPLL by age- and gender-stratified gross wages from age of death until 64 (Additional file 1). Estimates were adjusted for unemployment and labour force participation rates. For example, a 40 year old female in 2009 had a 0.69 probability of participating in the work force and a 0.93 probability of being employed if participating. These probabilities were applied to her assumed annual wage rate of €37,140 in 2009 (i.e. (37,140*0.69)*0.93)). The effect of age on wages as individuals transition to different wage categories based on hypothetical age progression was accounted for, as was labour force participation and employment progression. Wage growth was calculated at 2.6% per annum [16] and a discount rate of 4% annually was applied [19]. Cost estimates were subsequently summed over deaths in each 5-year age group to yield age group totals and across age groups to provide totals for all cancers combined and each cancer site. Premature mortality costs were also expressed per cancer death and per YPPLL.

In calculating the value of lost production from non-market activities, household activity was valued at €15.36 per hour and caregiving activity at €16.82 per hour. Time spent on each activity was multiplied by these wage rates, aggregated to an annual cost and summed over working life expectancy.

### Sensitivity analyses

We investigated the sensitivity of the base-case estimates to variations in several parameters. The wage growth rate was varied to 1.5% and 3.5% to account for uncertainty over future growth in the Irish economy. The discount rate was varied to 2% and 6%. More up-to-date estimates of labour force participation rates and employment rates were used to account for changing labour market conditions [15]. In addition the effect of extending the retirement age from 65 to 66 was explored, to account for a change (to be implemented in 2014) in the official pension age in Ireland.

## Results

### Total deaths, world age-standardised mortality rates (WASRs) and YPPLL by gender

Table 1 presents the number of deaths in people of all ages, WASRs and YPPLL for all cancers and by site, for males and females. The top 10 ranked cancers accounted for 77% of this total in males and 75% in females. Lung cancer was the most common cause of cancer-related death in males; breast cancer in females. WASR rankings by cancer site were relatively consistent with these rankings.

**Table 1:** Average annual number of deaths, WASRs and YPPLL for the 10 most common male and female cancers, and all cancers, in Ireland (2005 - 2009)

**ICD10**	**Cancer site**	**All deaths**	**% of the total**	**WASR per 100,000**	**% of the total**	**YPPLL**	**% of the total**
**Males**							
**C00-97,B21**	**All cancers**	**4,276**	**-**	**140.0**	**-**	**10,873**	**-**
	**Top 10 cancers**	**3,272**	**76.5**	**-**	**-**	**7,453**	**68.5**
C15	Oesophagus	218	5.1	7.4	5.3	598	5.5
C16	Stomach	207	4.8	6.8	4.9	550	5.1
C18-21	Colorectal	554	13.0	17.9	12.8	1,211	11.1
C25	Pancreas	217	5.1	7.4	5.3	603	5.5
C33-34	Lung	994	23.4	33.0	23.6	2,089	19.2
C61	Prostate	530	12.4	15.6	11.1	207	1.9
C67	Bladder	121	2.8	3.7	12.6	151	1.4
C70-72	Brain & CNS	150	3.5	5.6	74.0	1,027	9.4
C82-85,C96	Non-Hodgkin’s lymphoma	132	3.1	4.4	3.1	520	4.8
C91-95	Leukaemia	150	3.5	5.0	3.6	499	4.6
**Females**							
**C00-97, B21**	**All cancers**	**3,791**	**-**	**104.1**	**-**	**12,119**	**-**
	**Top 10 cancers**	**2,860**	**75.4**	**-**	**-**	**9,595**	**79.2**
C15	Oesophagus	118	3.1	2.8	2.7	178	1.5
C16	Stomach	130	3.4	3.3	3.2	334	2.8
C18-21	Colorectal	401	10.6	10.0	9.6	856	7.1
C25	Pancreas	218	5.8	5.5	5.3	361	3.0
C33-34	Lung	668	17.6	18.6	17.9	1,542	12.7
C50	Breast	679	17.9	20.5	19.7	3,329	27.5
C53-55	Uterus	172	4.5	5.5	5.3	1,126	9.3
C56	Ovary	254	6.7	7.7	7.4	901	7.4
C70-72	Brain & CNS	109	2.9	3.6	3.5	683	5.6
C82-85,C96	Non-Hodgkin’s lymphoma	111	2.9	2.8	2.7	288	2.4

Bold refer to the sum aggregated totals for ‘all cancers’ and the ‘top 10 cancers’ ranked in Ireland by mortality.

The total YPPLL for all cancers combined was 10% lower for males than females (10,873 vs 12,119). For some cancers, their YPPLL ranking differed from their rankings according to numbers of deaths. For example, in males, brain & CNS cancers ranked 7^th^ in terms of deaths and 3^rd^ in YPPLL while prostate ranked 3^rd^ in deaths and 9^th^ for YPPLL.

### Base-case analyses: total premature mortality costs, overall and by gender

**Figure 1:**
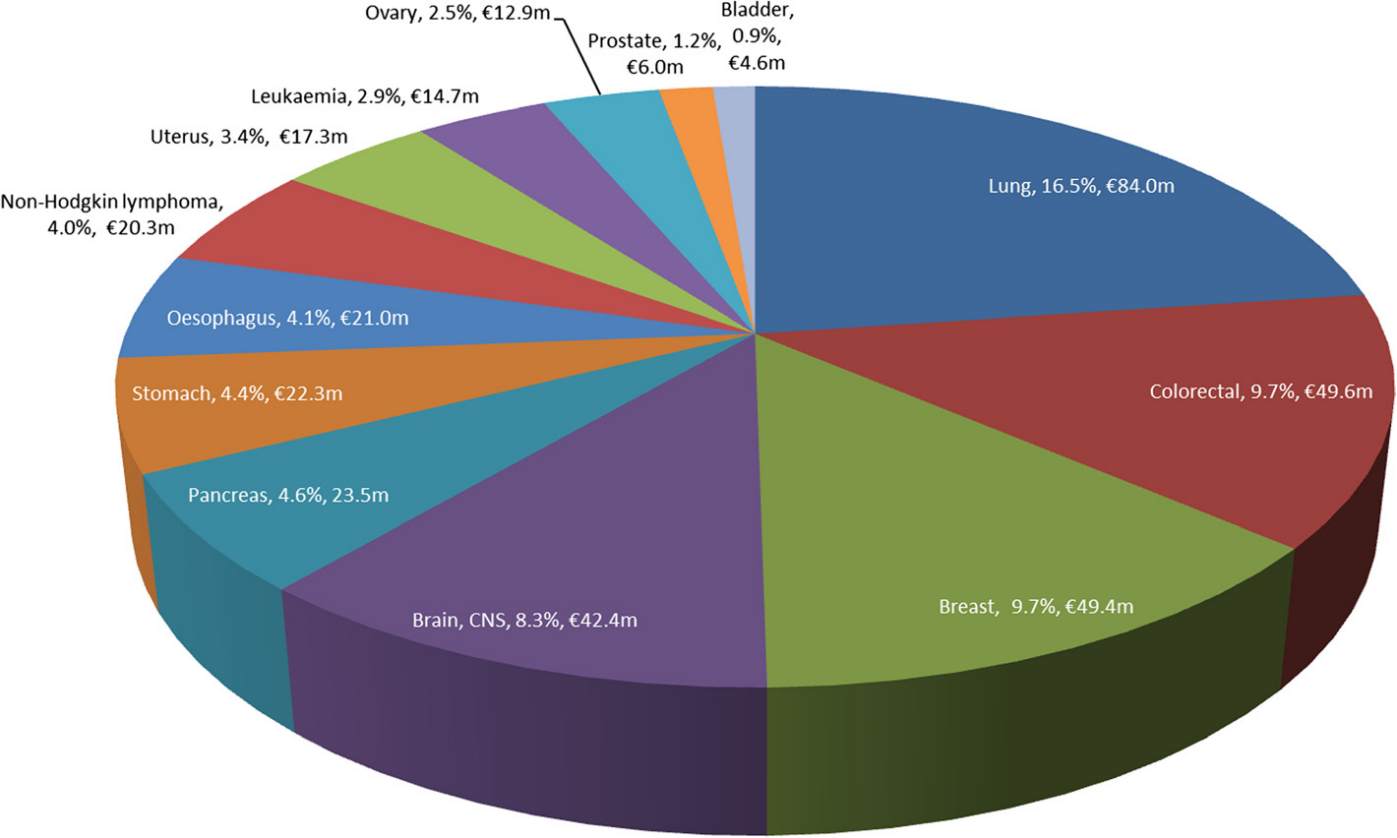
Percentage and value of total premature mortality costs (€, 2009) for male and female cancers combined.

Together, in the base-case analysis, all cancer sites generated a total of €509.5 million in premature mortality costs in 2009 (Table 2). In both sexes combined, lung cancer accounted for 16.5% (€84.0 million) of overall costs (Figure 1). This was followed by colorectal cancer (9.7%; €49.6 million), female breast cancer (€49.4 million; 9.7%) and cancers of the brain & CNS (€42.4 million; 8.3%).

**Table 2:** Premature mortality costs (€, 2009) for the 10 most common male and female cancers, and all cancers, in Ireland

**ICD10**	**Cancer site**	**Total premature mortality cost**	**% of the total**	**Premature mortality cost per death**	**Premature mortality cost per YPPLL**
**Males**					
**C00-97,B21**	**All cancers**	**332,246,992**	**-**	**290,172**	**30,558**
	**Top 10 cancers**	**228,125,322**	**68.7**	**276,918**	**31,381**
C15	Oesophagus	18,564,734	5.6	268,276	31,045
C16	Stomach	17,294,647	5.2	315,596	31,445
C18-21	Colorectal	37,253,268	11.2	276,770	30,775
C25	Pancreas	18,624,081	5.6	263,797	30,911
C33-34	Lung	62,927,480	18.9	220,953	30,123
C61	Prostate	5,950,929	1.8	154,169	28,818
C67	Bladder	4,637,827	1.4	246,693	30,714
C70-72	Brain & CNS	32,011,173	9.6	421,200	32,367
C82-85,C96	Non-Hodgkin’s lymphoma	16,113,046	4.8	385,480	36,579
C91-95	Leukaemia	14,748,138	4.4	426,247	34,100
**Females**					
**C00-97,B21**	**All cancers**	**177,266,357**	**-**	**159,959**	**14,628**
	**Top 10 cancers**	**139,871,322**	**78.9**	**155,275**	**14,578**
C15	Oesophagus	2,442,679	1.4	118,577	13,762
C16	Stomach	4,985,032	2.8	178,037	14,925
C18-21	Colorectal	12,393,845	7.0	147,195	14,487
C25	Pancreas	4,847,345	2.7	105,377	13,428
C33-34	Lung	21,099,185	11.9	114,919	13,683
C61	Breast	49,395,514	27.9	172,350	14,840
C67	Uterus	17,291,434	9.8	208,331	15,363
C70-72	Ovary	12,862,247	7.3	141,343	14,283
C82-85,C96	Brain & CNS	10,370,158	5.9	202,542	15,183
C91-95	Non-Hodgkin’s lymphoma	4,183,884	2.4	157,289	14,553

Bold refer to the sum aggregated totals for ‘all cancers’ and the ‘top 10 cancers’ ranked in Ireland by mortality.

The total all cancers premature mortality cost was 1.9 times higher in males than females (€332.2 million vs €177.3 million; Table 2). Lung cancer was the most expensive male cancer costing €62.9 million (19% of total male cancer costs). The most expensive female cancer was breast (€49.4 million; 28% of total female costs).

### Premature mortality cost per death and per YPPLL

The average male premature mortality costs per cancer death was 81% higher than the equivalent female cost (€290,172 vs €159,959; Table 2). Among the top 10 sites, in males, the most costly cancer per death was leukaemia (€426,247 per death); prostate cancer was the least costly (€154,169). The highest female cost per death was for cancer of the uterus (€208,331) and the lowest for pancreatic cancer (€105,377).

In males, for all cancers combined, the cost per YPPLL was €30,558, 109% higher than the equivalent female cost (€14,628). Male costs per YPPLL ranged between €28,818 (prostate) and €36,579 (Non-Hodgkin’s lymphoma) and female costs ranged between €13,428 (pancreas) and €15,363 (uterus).

### Gender distribution of YPPLL and premature mortality costs by age

**Figure 2:**
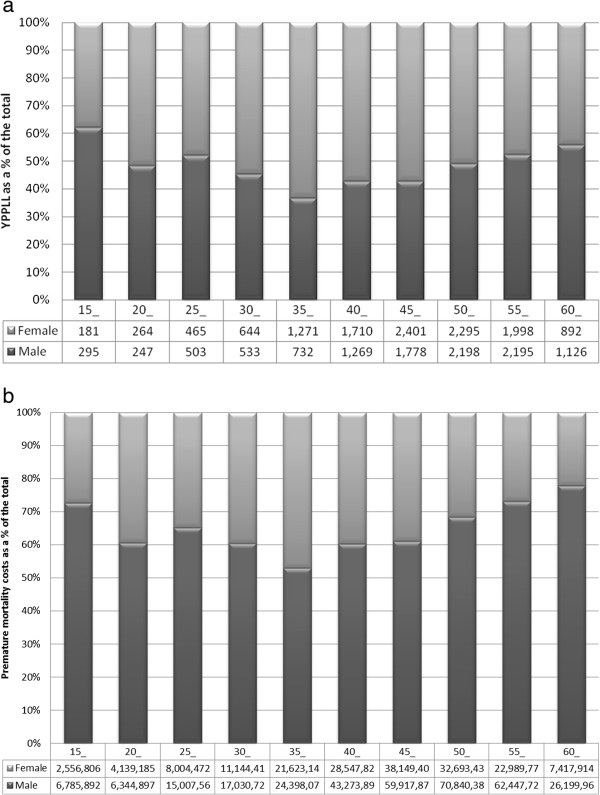
YPPLL and total premature mortality costs results by age. (a) **YPPLL and total premature mortality costs results by age. (a)**: Percentage of total YPPLL in males and females^1^, by age-group, for all cancers. ^1^The figures below the bars are the number of YPPLL in males and females in each age-group. **(b)**: Percentage of total premature mortality costs in males and females^1^, by age-group, for all cancers. ^1^The figures below the bars are the premature mortality costs (€2009) in males and females in each age-group.

Figures 2a and b show, by sex, the distribution of YPPLL and premature mortality costs for all cancers combined by 5-year age-group. While YPPLL were higher in females than males between 30 and 49 years, premature mortality costs for males exceeded those for females across all age groups with the differential between the sexes increasing with age after 45.

### Sensitivity analysis

Table 3 presents the results of the sensitivity analysis. Varying the discount rate had the greatest impact on the estimate of all-cancer premature mortality costs. With a lower discount rate, the total cost was 15-16% higher in both sexes; with a higher rate, it was 12% lower. An assumption of lower wage growth resulted in a 7-8% lower cost in both sexes, and higher growth in a 6-7% higher cost. Accounting for recent labour market conditions had a greater impact on male costs (+11%) than female costs (+1%). An extension of the retirement age by 1 year to 66 resulted in increased costs by 10% in males and 6% in females.

**Table 3:** Sensitivity analyses for all site cancer premature mortality costs (€, 2009) according to different assumptions for the discount rate, wage growth, labour market characteristics and the retirement age

	**Male**			**Female**		
	**Total premature mortality cost**	**% change from BC**	**Premature mortality cost per death**	**Total premature mortality cost**	**% change from BC**	**Premature mortality cost per death**
**BC**	**332,246,992**	**-**	**290,172**	**177,266,357**	**-**	**159,959**
**Wage growth (BC: 2.6%)**						
***1.50%***	307,469,158	(-7.5)	268,532	164,171,979	(-7.4)	148,143
***3.50%***	353,158,857	(+6.3)	308,436	189,439,743	(+6.9)	170,944
**Discount rate (BC: 4%)**						
***2%***	384,579,486	(+15.8)	335,877	206,654,039	(+16.6)	186,477
***6%***	291,598,265	(-12.2)	254,671	155,339,730	(-12.4)	140,173
**Labour force participation & unemployment (BC: 2009 rates)**						
***2013 rates***	368,598,902	(+10.9)	321,920	178,709,602	(+0.8)	161,261
**Retirement age (BC: 65)**						
***Age = 66***	365,157,324	(+9.9)	318,915	187,656,321	(+5.9)	169,334

BC: Base-case.

### Scenario analysis: Lost household production and caring activity

For all cancers, the total cost of lost household production and caring activities was €107.6 m for males and €328.7 m for females (Table 4). Male costs were €68.1 m for lost household production and €39.4 m for lost caregiving; female costs were €163.6 m and €165.1 m respectively. In females, the most costly site in terms of non-market activities was breast cancer (household production: €45.2 m; caregiving: €45.6 m). In males it was lung cancer (household production: €13.5 m; caregiving €7.8 m).

**Table 4:** Non-market - lost household production and caring activity - premature mortality costs (€, 2009) for the 10 most common male and female cancers, and all cancers, in Ireland

**ICD10**	**Cancer site**	**Total lost household production cost**	**Total lost caring cost**
**Male**			
**C00-97,B21**	**All cancers**	**68,119,367**	**39,440,641**
	**Top 10 cancers**	**47,031,364**	**27,230,833**
C15	Oesophagus	3,817,270	2,210,173
C16	Stomach	3,459,966	2,003,296
C18-21	Colorectal	7,646,872	4,441,163
C25	Pancreas	3,854,138	2,231,519
C33-34	Lung	13,500,891	7,816,922
C61	Prostate	1,361,957	788,564
C67	Bladder	964,857	558,646
C70-72	Brain & CNS	6,253,721	3,620,861
C82-85,C96	Non-Hodgkin’s lymphoma	3,186,066	1,844,710
C91-95	Leukaemia	2,962,003	1,714,979
**Female**			
**C00-97,B21**	**All cancers**	**163,592,354**	**165,057,650**
	**Top 10 cancers**	**130,228,532**	**131,394,989**
C15	Oesophagus	2,455,357	2,477,349
C16	Stomach	4,482,568	4,522,718
C18-21	Colorectal	11,646,872	11,751,193
C25	Pancreas	5,044,263	5,089,444
C33-34	Lung	21,421,357	21,613,229
C61	Breast	45,150,884	45,555,300
C67	Uterus	14,988,015	15,122,262
C70-72	Ovary	12,270,087	12,379,990
C82-85,C96	Brain & CNS	8,921,161	9,001,068
C91-95	Non-Hodgkin’s lymphoma	3,847,969	3,882,435

Bold refer to the sum aggregated totals for ‘all cancers’ and the ‘top 10 cancers’ ranked in Ireland by mortality.

## Discussion

### Cancer burden: the value of a range of perspectives

Premature mortality costs provide a different perspective of the cancer burden on society. They provide information that complements other more conventional indicators such as numbers of deaths or WASRs. For example, according to WASRs the all-sites cancer burden is somewhat higher in males than females (WASR M:F = 1.13). When YPPLL are considered the overall burden is somewhat lower in males (M:F = 0.89), due to the slightly older age distribution of deaths in males than females under 65. However, when considering premature mortality costs from lost market activities a different picture emerges: male productivity costs dwarf female costs (€332.2 million vs €177.3 million; M:F = 1.87). This is due to the incorporation of economic information such as labour force participation and wage rates into the estimates which tend to inflate male costs relative to female costs. For instance, the average male workforce participation rate in 2009 between 15 and 64 years was 77% compared to 60% for females, and the average male wage was €44,831 compared to €32,021 for females.

The calculation of premature mortality costs also revealed a new perspective on the relative importance of certain cancer sites within the overall cancer burden. For example, brain & CNS cancers emerged as a more important component of the cancer burden when premature mortality costs per death were considered (ranking 2^nd^ for males and 2^nd^ for females) rather than numbers of deaths (ranking 7^th^ and 10^th^). In contrast, prostate cancer dropped from 3^rd^ in terms of numbers of deaths to 9^th^ in terms of total premature mortality costs. These changes are driven by the difference between the distribution of the ages of death for individual cancers and the age of retirement: this is discussed further below.

### Economy-level premature mortality cost burden

The estimated premature cancer-related mortality productivity costs were substantial - €332.2 million for males and €177.3 million for females in 2009, resulting in a total annual cancer burden of over half a billion euros for Ireland. The limited previous literature in this area, primarily from the USA, also suggests that premature mortality productivity costs are considerable; representing approximately 1% of GDP [8]. Our total cost estimate for Ireland amounted to 0.3% of GDP. Our results also resonate on a cost per death basis. The estimates (€290,172 per cancer death among males and €159,959 per cancer death among females) are between 4 and 7 times the average wage in Ireland.

In the context of other cancer-related economic costs, it is worth noting that productivity costs are commonly reported to be far in excess of the direct medical costs of cancer treatment [5,7,20]. For example, in this study the premature mortality cost of colorectal cancer was €226,906 per death which is almost six times higher than the average cost of diagnosis, treatment and follow-up for a case of colorectal cancer in Ireland (€39,607 in 2008) [21]. Moreover, it is worth bearing in mind that premature mortality costs are only one element of the total productivity loss from market activities due to cancer; other elements include costs relating to absenteeism from work and reduced work ability due to cancer or its treatment [22].

In recognition of the fact that individuals also engage in a range of non-market activities, the loss of which through premature cancer-related mortality also represents an economic loss to society, we estimated costs of potential household production and caring activity lost. These estimates included the time lost by both working and non-working individuals and thus extended the perspective of the base-case analysis. A previous study that estimated the value of lost household production and caring activity due to premature mortality across multiple cancer sites reported a doubling of costs due to the addition of non-market activity lost to market-activity [8]. Our findings were similar; the all cancer total lost productivity cost increased from €509.5 m to €945.7 m when losses from non-market activities were added to market activities. As would be expected, due to fact that women generally undertake more household and caregiving activities than men, the influence of the inclusion of non-market activities was greater for females than males. Indeed, following the inclusion of non-market activity costs, total female costs surpassed total male costs overall (€506.0 m v €439.8 m).

### International comparison of premature mortality costs

Two studies have estimated the productivity costs associated with multiple cancer sites in the US. Applying the human capital approach, Bradley *et al*. [8] estimated US cancer-related productivity costs of $142.4 billion in 2010 with lung, colorectal and female breast cancer accounting for almost half (44%) of all costs. These findings correspond to the rankings in our study based on the costs of market activities, although the combined cost of the three cancer sites in Ireland was somewhat less as a proportion of the total (36%). This difference is due to a greater proportion of deaths in Ireland due to breast cancer (Ireland: 12.7% vs US: 7.4%) compared to lung cancer (Ireland: 20.8% vs US: 28.2%) and lower labour force participation rates for females in Ireland compared to the US.

In the US, testicular cancer ranked as the most expensive site ($1,267,803 per cancer death) followed by Hodgkin’s lymphoma ($544,118) and brain & CNS ($392,853). We did not include testicular cancer and Hodgkin’s disease since they ranked outside the ten most common causes of cancer-related death, but did consider brain & CNS cancers. After accounting for the exchange rate, the premature mortality costs of brain & CNS cancer was somewhat higher in Ireland than the US (€420,160 vs €296,622). The difference may be due to the truncation our estimates at 65 years (the retirement age in Ireland), while the US study included deaths of all ages.

Few other studies are available for comparative purposes. In Korea [10], liver and stomach cancer emerged as the largest contributors to total cancer-related premature mortality costs based on a relatively higher number of deaths from these cancers than in Ireland or the US (Korea - stomach cancer: 18.7% of total cancer deaths; liver cancer: 17.7%).

While these comparisons suggests a (modest) degree of consistency internationally with regard to the premature mortality cost burden, they equally highlight the importance of estimating costs specific to an individual setting, in order to account for geographical differences in the pattern of cancer deaths and labour force dynamics.

### Individual cancer sites

While the observation that lung cancer was the most costly cancer overall in the base-case analyses is unsurprising (given its high relative mortality in both genders in Ireland), the emergence of cancers of the brain & CNS as the second most expensive in terms of cost per death in males and females is more interesting. For cancers of the brain & CNS, age of diagnosis is relatively low (median = 57 vs 67 for all cancers) [23] as is survival (5-year relative survival for cases diagnosed 2003-2007 was less than 20%) [24]: hence more than 40% of deaths occur in people younger than retirement age. This reveals how numbers of deaths, age at diagnosis and survival all impact on cost per death: in particular, cancers with earlier age at onset and which have moderate or poor prognosis, tend to rank more highly in terms of cost per death.

Other cancers also ranked differently when alternative metrics were compared. For example, prostate cancer accounted for 12.4% of all male cancer deaths but was one of the lower cost cancers both in terms of total cost and cost per death. This was due to the relatively low proportion of deaths in men of working-age combined with high survival (almost 90% 5-year relative survival) [25]. Indeed, while 37% of men diagnosed with prostate cancer were under 65 years at diagnosis, only 7% of deaths from prostate cancer were in this age group [25].

### Implications: value of estimates of lost productivity costs

Estimates of the size of the monetary burden associated with premature mortality due to cancer may assist policymakers in deciding the allocation of funds among competing cancer control activities [8]. In particular they could help form a picture of the reduction in the economic burden that may be achieved by implementation of particular primary, secondary or tertiary prevention strategies. As previous research has noted failure to include these costs in decision-making leads to an underestimation of societal costs and may lead to welfare damaging decisions [26]. Estimates of productivity losses may also be important sources of data for economic evaluations of specific health technologies. Some leading expert panels (for example the US Panel on Cost-Effectiveness in Health and Medicine [27]) and economic textbooks and advocate a societal perspective for economic evaluations but, in reality, relatively few evaluations include productivity costs [26], perhaps due to a lack of available data (in some instances, at least). Moreover, combined with direct medical costs and direct non-medical costs (including patient time, travel and out-of-pocket costs), such estimates provide an important - yet rarely quantified - building block in constructing an accurate measure of the total economic burden of cancer on society.

### Strengths and limitations

As far as we are aware, this study provides the first estimates of lost productivity costs for multiple cancer sites for a western European country. Key strengths of the study include the use of population data and the application of a simple and transparent methodology to value lost productivity. Nevertheless, there are some limitations. Use of the human capital approach to estimate costs for market activities puts greater weight on older males who are working compared to younger citizens (who earn lower wages), especially younger females. For example a male aged 50 earned €55,967 on average in 2009, whereas a female aged 30 earned €37,549. Nevertheless, the human capital approach is widely used throughout the economic literature thus enhancing the comparability of our results. Alternative methodological techniques including the friction cost approach and the willingness-to-pay approach are also not without flaws and limitations [11].

In terms of potential limitations, our estimates for lost household production and caring activity were based on reported time use in 2005 in the general population as no equivalent estimates exist for the cancer population. If patterns of time use in cancer patients differ from those among people without cancer (which they might well do due to the effects that cancer and its treatment may have on functional status) our estimates will not reflect the true value of this potential lost non-market activity. Uncertainty also remains over the correct valuation method for non-market activity. While we used the proxy good approach in this study, other methods exist and these may result in quite different estimates [12].

We did not estimate productivity losses due to morbidity; this was due to a lack of reliable site-specific data. However, most previous studies have found that premature mortality costs constitute the overwhelming majority of the total productivity cost for market activities [6,7,28,29]. We focused on the potential productivity losses to the economy of working individuals between the ages of 15 and 64: non participants in the labour force were thus explicitly excluded in the base-case analysis. The choice of 64 as a cut-off age reflected the official pensionable age in Ireland in 2009 beyond which the majority of individuals retire. While the effective retirement age can be lower (or higher) than the pensionable age, in Ireland the effective retirement age is 64.6 for males and 62.6 for females (http://www.oecd.org/els/public-pensions/ageingandemploymentpolicies-statisticsonaverageeffectiveageofretirement.htm).

## Conclusion

Lost productivity costs due to cancer-related premature mortality are significant, amounting to over half a billion euros in Ireland in 2009. Total lost productivity costs due to market activities were 1.65 times higher in males than females, due to higher wages and higher rates of workforce participation. Lung and breast cancer – but not prostate cancer - were major contributors to the overall total cost. Cancers with earlier age at onset and lower survival, ranked more highly as regards cost per death. Productivity costs provide an alternative perspective of the cancer burden on society. They give an indication of the potential cost savings that could accrue from effective primary prevention, earlier diagnosis and/or advances in treatments, and may assist policymakers in determining allocation of funds among competing health care interventions, especially during times of constrained finances.

## Abbreviations

YPPLL: Years of potential productive life lost; WASRs: World age-standardised mortality rates; GDP: Gross domestic product.

## Competing interests

The authors declare that they have no competing interests.

## Authors’ contributions

PH contributed to the conception and design of the study, the acquisition of mortality and economic data, and the calculation of YPPLL and productivity costs. LS contributed to the conception and design of the study, the acquisition of mortality data, and the calculation of WASRs. Both authors were involved in drafting and revising the article, and both have read and approved the final manuscript.

## Pre-publication history

The pre-publication history for this paper can be accessed here:

http://www.biomedcentral.com/1471-2407/14/224/prepub

## Supplementary Material

Additional file 1 The cost of lost productivity due to premature cancer-related mortality: an economic measure of the cancer burden. Click here for file
