# Supplementary material for: The cost of lost productivity due to premature cancer-related mortality: an economic measure of the cancer burden
Source: BMC Cancer. 2014 Mar 26;14:224. doi: 10.1186/1471-2407-14-224 (PMC3986872; doi:10.1186/1471-2407-14-224)

**Supplementary Material: The cost of lost productivity due to premature cancer-related mortality: an economic measure of the cancer burden**

**Figure 1A: Male wage rate transition between ages 15-64 for 2009**


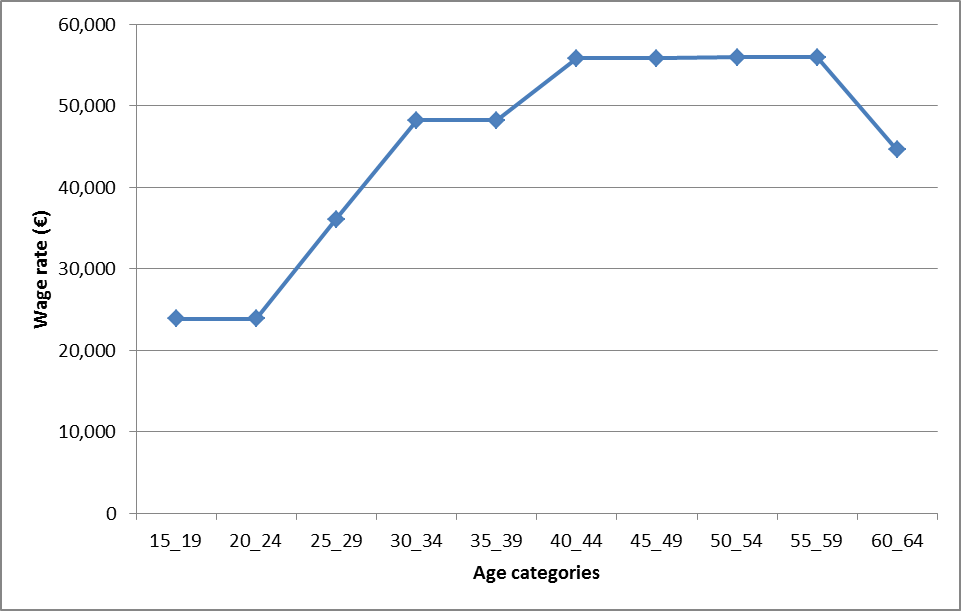


**Figure 2A: Female wage rate transition between ages 15-64 for 2009**


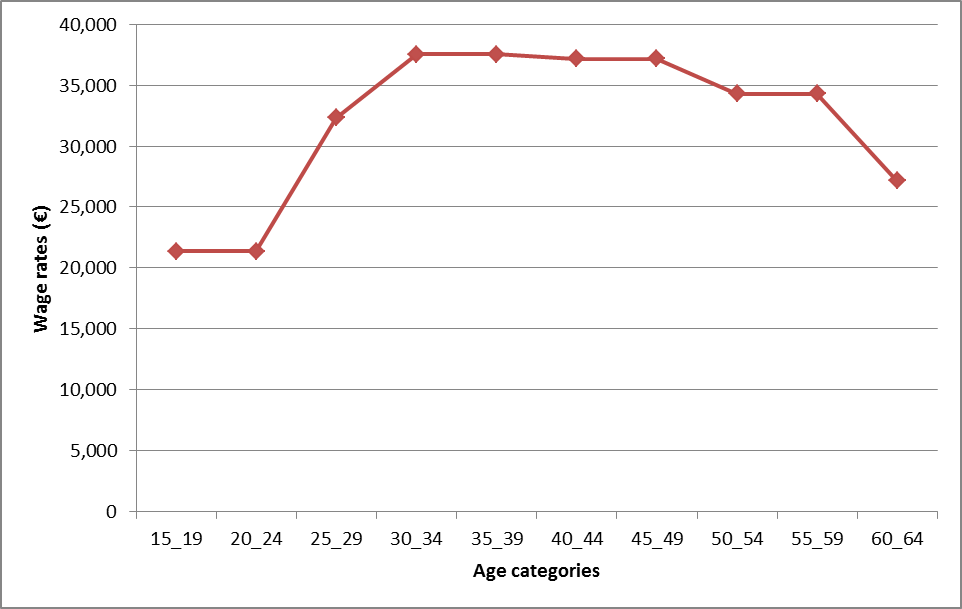

Supplement: Additional file 1 — The cost of lost productivity due to premature cancer-related mortality: an economic measure of the cancer burden. [file 1471-2407-14-224-S1.docx]
